# Pretreatment CONUT Score and Overall Survival in Patients with Metastatic Pancreatic Adenocarcinoma Receiving First-Line Chemotherapy: A Retrospective Cohort Study

**DOI:** 10.3390/medicina62091732

**Published:** 2026-09-09

**Authors:** Zeynep Alaca Topcu, Serhat Demirer, Tuba Baydas, Mehmet Besiroglu, Mahmut Gumus

**Affiliations:** 1 Department of Medical Oncology, Goztepe Prof. Dr. Suleyman Yalcın City Hospital, Istanbul Medeniyet University, İstanbul 34722, Türkiye; tuba.baydas@gmail.com (T.B.); mbesiroglu@yahoo.com.tr (M.B.); mgumus@superonline.com (M.G.); 2 Department of Medical Oncology, Haydarpasa Numune Research and Training Hospital, İstanbul 34668, Türkiye; dr.serhatdemirer@gmail.com

**Keywords:** CONUT score, malnutrition, metastatic pancreatic cancer, overall survival

## Abstract

*Background and Objectives*: Metastatic pancreatic adenocarcinoma is related to poor prognosis and is frequently accompanied by malnutrition, inflammation, and immune dysregulation. The Controlling Nutritional Status (CONUT) score is a composite laboratory-based index that may reflect aspects of nutritional and immune status. This study aimed to evaluate the association between pretreatment CONUT score and overall survival (OS) in patients with metastatic pancreatic adenocarcinoma receiving first-line systemic chemotherapy. *Materials and Methods*: In this retrospective analysis, a total of 156 patients diagnosed with metastatic pancreatic adenocarcinoma between January 2017 and July 2024 and treated with first-line systemic chemotherapy were included. Based on the conventional CONUT classification, patients were categorized a priori into normal-CONUT (scores 0–1) and elevated-CONUT (scores ≥ 2) groups, with CONUT additionally evaluated as a continuous score. OS was estimated using the Kaplan–Meier method. Univariable and multivariable Cox regression analyses were performed to evaluate the association between the CONUT score, selected clinical factors, and overall survival. *Results*: The cohort demonstrated a median OS of 9.1 months (95% CI: 6.6–11.6). OS was significantly better among individuals classified in normal CONUT group relative to elevated CONUT group (14.1 vs. 6.5 months, respectively, *p* <0.001). Higher continuous CONUT scores were also associated with shorter OS (HR 1.23 (1.14–1.32), *p* < 0.001). In multivariable analysis, an elevated CONUT score (HR, 2.40 (1.63–3.52); *p* <0.001), liver metastases (HR 1.99 (1.31–3.02); *p* = 0.001), and the presence of comorbidity (HR, 1.88 (1.28–2.75); *p* = 0.001) remained significantly associated with shorter OS. *Conclusions*: An elevated pretreatment CONUT score was associated with shorter overall survival in this selected chemotherapy-treated cohort. As a readily available laboratory-based index, CONUT may provide additional prognostic information; however, prospective studies are needed to determine whether it adds value beyond established clinical factors.

## 1. Introduction

Pancreatic cancer remains one of the most lethal malignancies worldwide. Although it ranks 12th in global cancer incidence, it is the seventh leading cause of cancer-related death [1,2]. Although important progress has been achieved in diagnostic modalities, surgical management, and systemic treatments, Pancreatic cancer continues to be associated with unfavorable prognosis, with a 5-year overall survival rate of nearly 12–13%. The unfavorable outcome is primarily attributed to the aggressive biology of the disease, early metastatic spread, as diagnosis commonly occurs during advanced-stage disease when curative surgical resection is no longer feasible [3,4]. Emerging evidence indicates that nutritional impairment and systemic inflammatory processes are strongly associated with tumor progression and survival outcomes in cancer patients. Malnutrition is highly prevalent among patients with pancreatic cancer due to cancer-related cachexia, metabolic alterations, and reduced oral intake. In this context, several nutritional and immunological indices have been developed to determine the inflammatory and nutritional condition of patients and to estimate prognosis in various malignancies. Current prognostic factors in this setting are insufficient to correctly stratify and characterize this population. The Controlling Nutritional Status (CONUT) score, which is determined using serum albumin levels, total cholesterol concentration, and lymphocyte count, has emerged as a practical and objective indicator reflecting both immune competence and nutritional status. The prognostic relevance of the CONUT score has been demonstrated in several cancers in prior studies, including gastric, colorectal, and hepatocellular carcinoma [5].

A recent umbrella review further supported the prognostic value of CONUT across various cancer types [6]. Although the prognostic relevance of CONUT has been evaluated in pancreatic cancer, including in a previous meta-analysis [7], evidence focusing specifically on patients with metastatic pancreatic adenocarcinoma receiving first-line systemic chemotherapy in routine clinical practice remains limited. Therefore, this retrospective study aimed to evaluate the association between pretreatment CONUT score and OS in this population.

## 2. Materials and Methods

### 2.1. Study Design and Patients

**Figure 1:**
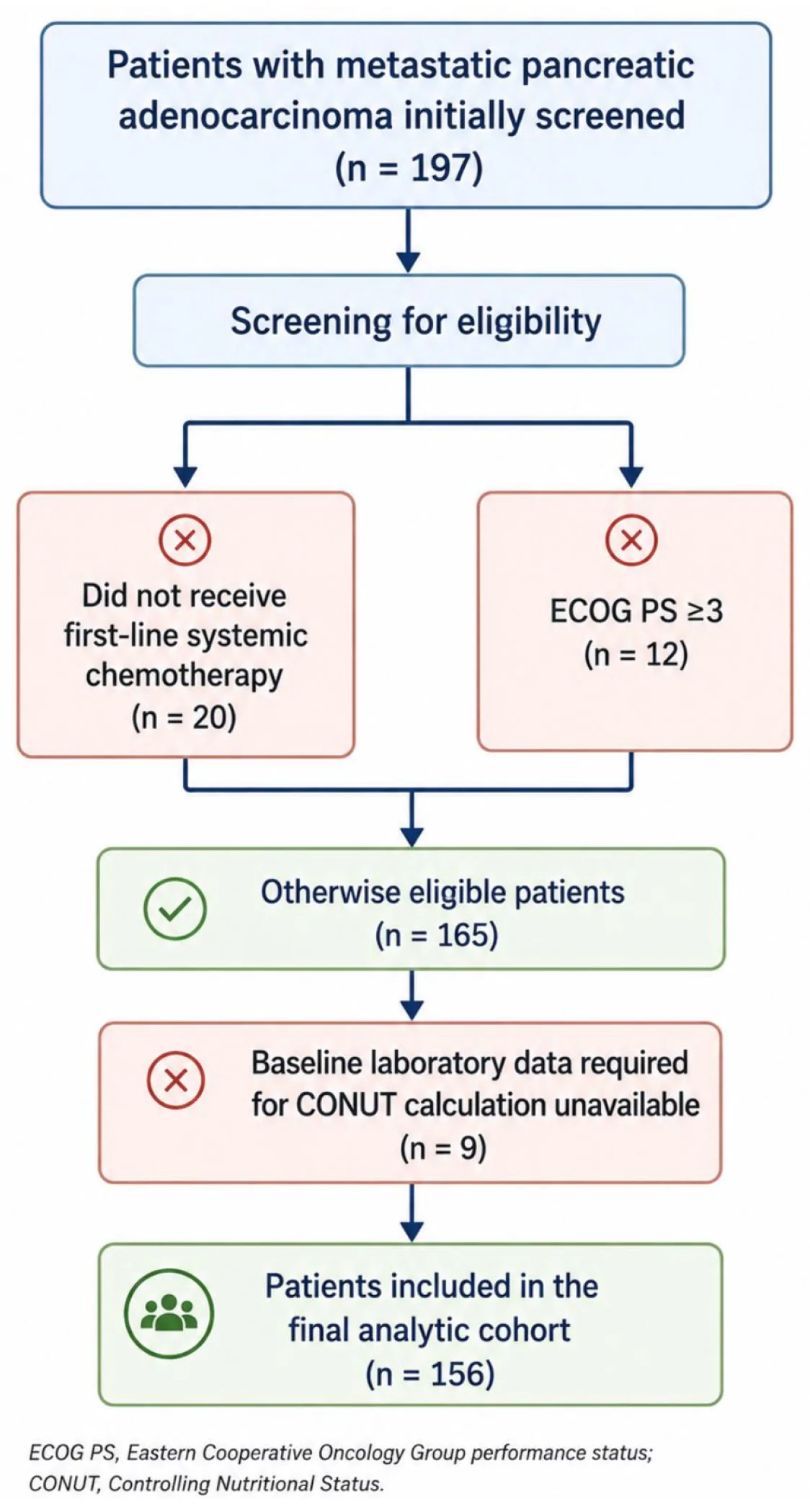
Patient selection flow diagram.

This retrospective cohort study included patients treated between January 2017 and July 2024. All consecutive adult patients with histologically confirmed pancreatic adenocarcinoma and radiologically confirmed metastatic disease, either at initial diagnosis or following recurrence after prior curative-intent surgery, were retrospectively screened for eligibility. Patients were eligible if they had received at least one cycle of standard first-line systemic chemotherapy for metastatic disease. The study population was restricted to patients who initiated first-line systemic chemotherapy because the predefined objective was to evaluate the prognostic association of pretreatment CONUT within a treatment-eligible metastatic population. Patients who did not receive systemic treatment were not included, as they represent a clinically distinct population in whom poor performance status, rapid clinical deterioration, or other factors precluding treatment may themselves strongly influence survival. Patients aged < 18 years and those with an Eastern Cooperative Oncology Group performance status (ECOG PS) ≥ 3 were excluded. Patients with ECOG PS ≥ 3 were excluded because they generally represent a poor-prognosis population with limited suitability for systemic chemotherapy, whereas the predefined objective of the study was to evaluate the prognostic value of CONUT in a treatment-eligible population. A total of 197 patients with metastatic pancreatic adenocarcinoma were initially screened. During screening, 20 patients who did not receive first-line systemic chemotherapy and 12 patients with ECOG PS ≥ 3 were excluded according to the predefined eligibility criteria. In addition, baseline laboratory data required for calculation of the CONUT score were unavailable for 9 otherwise eligible patients; these patients could therefore not be included in the final analytic cohort. The final analytic cohort consisted of 156 patients. The patient selection process is summarized in Figure 1.

### 2.2. Patient Evaluation

Demographic, radiological, pathological, laboratory, and clinical information was retrospectively obtained from institutional medical records. Information regarding tumor characteristics, metastatic disease burden, treatment history, and baseline laboratory findings at the time of metastatic diagnosis was collected and evaluated. Comorbidity was defined as the presence of at least one documented diagnosis of diabetes mellitus, hypertension, or coronary artery disease at the time of metastatic disease diagnosis. Comorbidity status was categorized as present or absent; the number and severity of comorbid conditions were not considered, and no validated comorbidity index was used. Radiological assessment was performed using computed tomography imaging, and treatment responses were evaluated based on the Response Evaluation Criteria in Solid Tumors version 1.1. Tumor evaluations were performed approximately every 8–12 weeks according to routine practice. OS was established from the date of metastatic diagnosis until death from any cause or the last available follow-up visit. Progression-free survival (PFS) was defined as the time from treatment initiation to disease progression, death, or last follow-up. Patients without progression or death were censored at the last clinical or radiological assessment. Nutritional status was assessed at metastatic diagnosis according to the CONUT scoring system. Data on formal nutritional assessment and supportive interventions were not systematically available in the retrospective records and were therefore not included in the analyses. The study protocol was approved by the Haydarpaşa Numune Training and Research Hospital Non-Interventional Clinical Research Ethics Committee (decision no. HNEAH-BAEK 2025/20; 25 February 2025). Due to the retrospective nature of the study, the requirement for written informed consent was waived by the ethics committee. The study was conducted in accordance with the principles of the Declaration of Helsinki.

### 2.3. CONUT Score Assessment

Baseline laboratory values were defined as the closest available measurements obtained within 30 days before initiation of first-line systemic chemotherapy. The CONUT score is calculated based on serum albumin level, total lymphocyte count, and total cholesterol level (5). The total CONUT score was calculated as the sum of the points obtained from these three parameters. Through the total CONUT score, participants were classified into four nutritional risk categories: normal nutritional status (0–1), mild malnutrition (2–4), moderate malnutrition (5–8), and severe malnutrition (9–12). Detailed components and the CONUT-based scoring criteria are summarized in Table 1.

### 2.4. Statistical Analysis

Statistical analyses were performed using the Statistical Package for the Social Sciences (SPSS) version 24.0 (IBM Corp., Armonk, NY, USA). Categorical variables were summarized as frequencies and percentages, while continuous and ordinal variables were expressed as mean ± standard deviation, median, and range. The Kolmogorov–Smirnov test was used to assess the normality of data distribution. The Pearson chi-square (χ^2^) test was applied to compare categorical variables. Patients’ clinical characteristics were evaluated using descriptive statistical analyses. Based on the conventional CONUT classification, patients were categorized a priori into a normal-CONUT group (scores 0–1) and an elevated-CONUT group (scores ≥ 2), representing any degree of CONUT-defined nutritional impairment. In additional analyses, CONUT was also evaluated as a continuous variable across its full 0–12 range and according to the conventional four categories (0–1, 2–4, 5–8, and 9–12). The association of each one-point increase in CONUT with OS and PFS was assessed using Cox proportional hazards regression. OS across the four CONUT categories was evaluated using the Kaplan–Meier method and compared using the log-rank test. OS was evaluated using the Kaplan–Meier method and compared between groups using the log-rank test. Univariable and multivariable Cox proportional hazards regression analyses were performed to identify factors associated with overall survival. Variables with a *p*-value < 0.10 in univariable analysis were entered into the multivariable model. Age, sex, metastatic presentation (de novo vs. metachronous), and ECOG performance status were retained regardless of their univariable significance because of their clinical relevance. Given the limited number of patients within individual treatment groups, an exploratory analysis compared patients receiving gemcitabine plus nab-paclitaxel, the most frequently administered first-line regimen, with those receiving all other regimens combined. OS was compared using the Kaplan–Meier method and univariable Cox regression analysis. Statistical significance was defined as a *p*-value < 0.05.

## 3. Results

A total of 156 patients were included in present study. The mean age was 62.9 ± 10.5 years, and 57.7% of the cohort were male. An ECOG-PS of 0–1 was detected in 87.7% of patients. Body mass index (BMI) averaged 24.6 ± 5.0 kg/m^2^. Liver metastases were the most common metastatic site (74.4%), and 63.5% of patients were involved of more than one metastatic organ. Various first-line chemotherapy regimens were administered in the study cohort, reflecting real-world clinical practice. A median of 5.1 treatment cycles was administered (1–17) of first-line chemotherapy in the metastatic setting. Gemcitabine plus nab-paclitaxel to 35 patients (22.5%), FOLFIRINOX was administered to 32 patients (20.5%), FOLFOX/XELOX to 32 patients (20.5%), gemcitabine plus cisplatin to 18 patients (11.5%), and single-agent gemcitabine to 30 patients (19.2%). Other regimens, including single agent capecitabine and gemcitabine plus capecitabine, were administered to 9 patients (5.8%). During follow-up, 34.6% of patients proceeded to second-line systemic therapy. According to the conventional CONUT classification, 51 patients (32.7%) were included in the normal-CONUT group and 105 patients (67.3%) in the elevated-CONUT group. When further stratified into the conventional four CONUT categories, 51 patients (32.7%) had scores of 0–1, 68 (43.6%) had scores of 2–4, 34 (21.8%) had scores of 5–8, and 3 (1.9%) had scores of 9–12.

**Figure 2:**
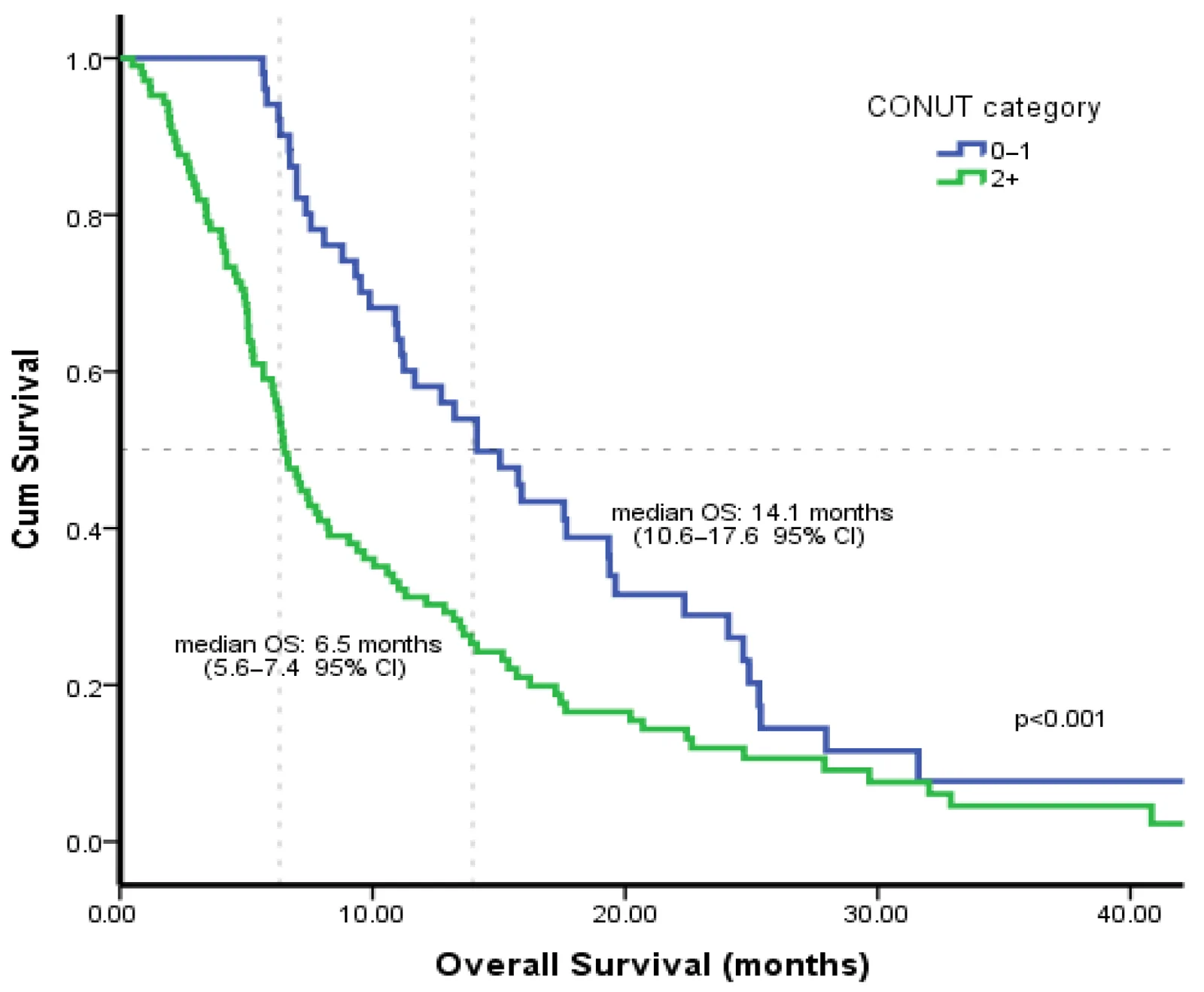
Kaplan–Meier–based overall survival evaluation across CONUT categories: normal CONUT (0–1) versus elevated CONUT (≥2).

**Figure 3:**
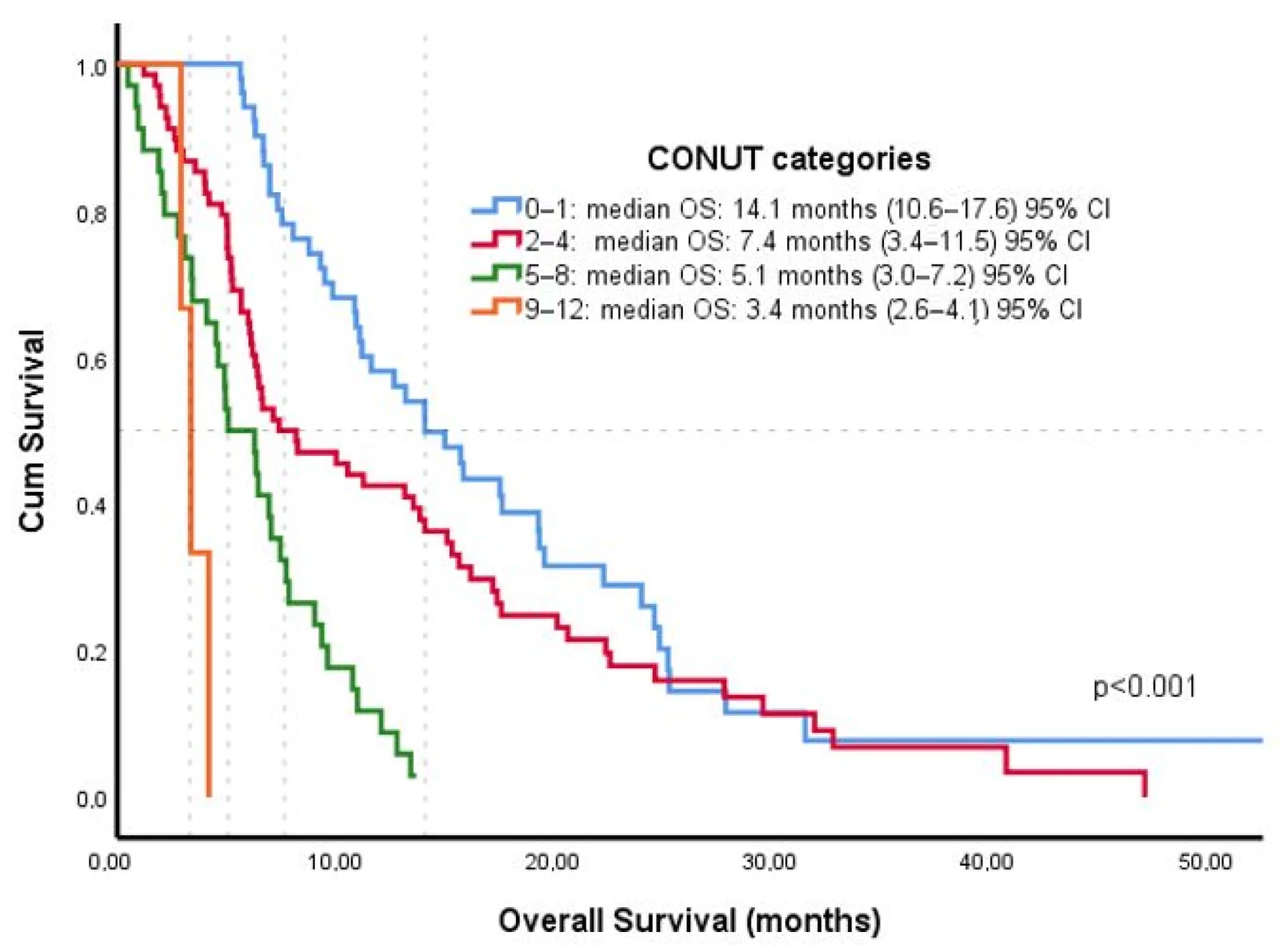
Kaplan–Meier analysis of overall survival according to the four conventional CONUT categories.

The cohort demonstrated a median OS of 9.1 months (95% CI: 6.6–11.6). Individuals with normal CONUT score (0–1) had a significantly improved median OS relative to those with elevated CONUT score (≥2) [14.1 months (95% CI: 10.6–17.6) vs. 6.5 months (95% CI: 5.6–7.4), respectively; *p* < 0.001] (Figure 2). The estimated 1-year OS was 40.0% in the overall cohort, whereas it was 58.1% in normal CONUT group and 31.2% in elevated CONUT group (*p* < 0.001). Similarly, the estimated 2-year OS rate for the whole cohort was 17.5%, 28.9% in participants with normal CONUT scores and 12.0% in those with high scores, demonstrating significantly poorer survival outcomes in patients with elevated CONUT category (*p* < 0.001). At the data cutoff, 139 of 156 patients (89.1%) had died. When evaluated according to the conventional four CONUT categories, median OS progressively decreased with increasing CONUT severity: 14.1 months (95% CI, 10.6–17.6) for scores 0–1, 7.4 months (95% CI, 3.4–11.5) for scores 2–4, 5.1 months (95% CI, 3.0–7.2) for scores 5–8, and 3.4 months (95% CI, 2.6–4.1) for scores 9–12 (log-rank *p* < 0.001) (Figure 3). Consistently, when analyzed as a continuous variable, each one-point increase in CONUT was associated with shorter OS (HR, 1.23; 95% CI, 1.14–1.32; *p* < 0.001).

**Figure 4:**
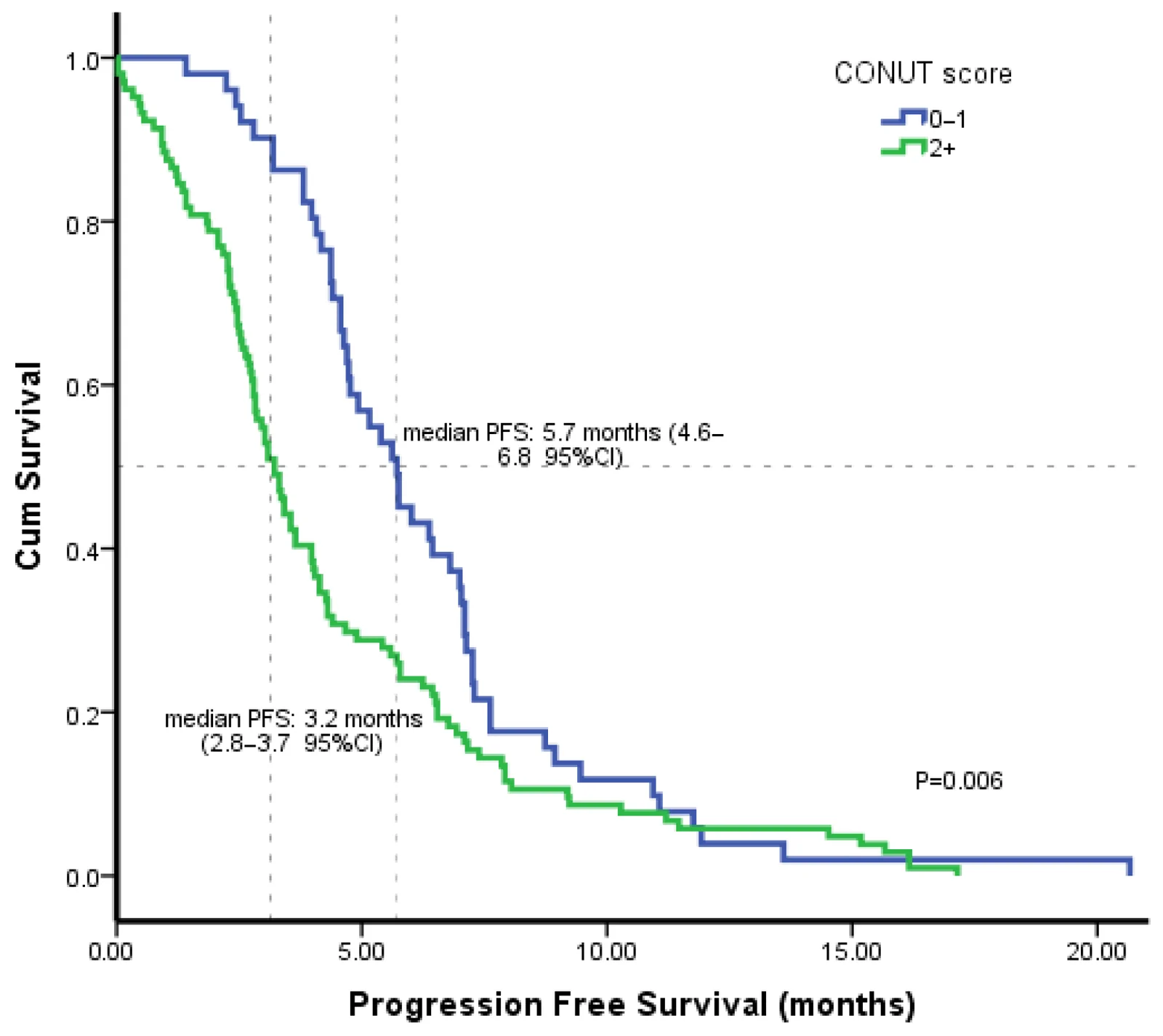
Progression-free survival based on CONUT status assessed by Kaplan–Meier analysis: normal CONUT (0–1) versus elevated CONUT (≥2).

The cohort demonstrated a median PFS of 4.1 months (95% CI: 3.6–4.7). Patients categorized as having normal CONUT scores showed significantly improved median PFS relative to patients with elevated CONUT scores [5.7 months (95% CI: 4.6–6.8) vs. 3.2 months (95% CI: 2.8–3.7), respectively; *p* = 0.006]. These survival differences are presented in Figure 4 When analyzed continuously, each one-point increase in CONUT was associated with shorter PFS (HR, 1.15; 95% CI, 1.08–1.24; *p* < 0.001).

Baseline demographic and clinicopathological characteristics according to CONUT groups are presented in Table 2. Baseline characteristics were generally comparable between the CONUT groups, except for weight-loss status.

In univariable Cox regression analysis, the existence of comorbidity (HR: 1.67 (1.15–2.43), *p* = 0.007), elevated CONUT score group (HR: 1.92 (1.33–2.77), *p* < 0.001), and liver metastases (HR: 1.61 (1.08–2.41), *p* = 0.015) were significantly related to overall survival. In the multivariable Cox regression analysis, elevated CONUT score remained significantly associated with shorter OS (HR, 2.40; 95% CI, 1.63–3.52; *p* < 0.001). Liver metastases (HR, 1.99; 95% CI, 1.31–3.02; *p* = 0.001) and the presence of at least one comorbidity (HR, 1.88; 95% CI, 1.28–2.75; *p* = 0.001) were also independently associated with shorter OS. Age, sex, ECOG PS, and metastatic presentation (de novo vs. metachronous) were not significantly associated with OS in the multivariable model. Given the substantial heterogeneity of first-line chemotherapy regimens and the limited number of patients within individual regimen groups, separate analyses for each treatment regimen were not considered appropriate. Therefore, an exploratory analysis compared gemcitabine plus nab-paclitaxel, the most frequently administered first-line regimen (n = 35), with all other regimens combined. Kaplan–Meier analysis showed no statistically significant difference in OS between patients receiving gemcitabine plus nab-paclitaxel and those receiving other regimens (median OS, 12.8 vs. 7.2 months, respectively; log-rank *p* = 0.144). In the univariable Cox regression analysis, receipt of other first-line regimens was not significantly associated with OS compared with gemcitabine plus nab-paclitaxel (HR, 1.33; 95% CI, 0.90–1.98; *p* = 0.145). Table 3 summarizes the results of the univariable and multivariable Cox regression analyses for overall survival.

## 4. Discussion

In this retrospective cohort of patients with metastatic pancreatic adenocarcinoma receiving first-line systemic chemotherapy, an elevated pretreatment CONUT score was associated with shorter OS and remained significantly associated after adjustment for selected clinical factors in the multivariable model. Liver metastases and the presence of comorbidity were likewise associated with poorer survival. By focusing on a clinically well-defined, chemotherapy-treated metastatic population managed in routine oncology practice, this study extends the available evidence on the prognostic relevance of CONUT in pancreatic cancer.

Metastatic pancreatic adenocarcinoma is associated with poor prognosis and limited survival outcomes and is frequently accompanied by profound malnutrition, cachexia, inflammatory activation, and immune dysfunction. Cancer-associated malnutrition and cachexia are particularly prevalent especially among patients with advanced-stage metastatic pancreatic cancer due to multiple mechanisms, including reduced oral intake, pancreatic exocrine insufficiency, tumor-associated catabolism and chronic systemic inflammation. Previous studies have shown that nutritional deterioration adversely affects treatment tolerance, quality of life, functional capacity, and survival results in the pancreatic cancer population. In addition, systemic inflammation and immune suppression are increasingly recognized as major contributors to tumor progression and resistance to therapy. Therefore, biomarkers integrating nutritional and immunological status may provide more comprehensive prognostic information than individual laboratory parameters alone [8,9,10]. A recent systematic review also supported the prognostic relevance of nutritional assessment tools, including CONUT, in patients with pancreatic cancer [11]. The CONUT score combines serum albumin, peripheral lymphocyte count, and total cholesterol, which may reflect overlapping nutritional, inflammatory, immune, and disease-related processes. Hypoalbuminemia may be associated with both nutritional impairment and systemic inflammation, whereas lymphopenia may reflect impaired immune status. Similarly, reduced cholesterol levels may be related to altered metabolism or reduced nutritional reserve. However, these components are not specific to nutritional status. Therefore, the association observed in our cohort should not be interpreted as evidence that nutritional impairment itself caused shorter survival [12,13,14].

The prognostic utility of the CONUT score has been documented in several cancers in prior reports, including gastric, colorectal, hepatobiliary, lung, and urothelial cancers [15,16,17,18,19]. The potential prognostic relevance of CONUT in pancreatic cancer has also attracted growing interest. A recent study also demonstrated the prognostic relevance of CONUT in patients with pancreatic cancer undergoing pancreatectomy [20]. In addition, Uemura et al. reported that higher CONUT categories were associated with shorter PFS and OS in patients with unresectable advanced pancreatic cancer receiving multi-agent chemotherapy [21]. More recently, Peker et al. also reported an association between higher CONUT scores and shorter OS in patients with metastatic pancreatic cancer [22]. Rather than introducing a new prognostic concept, the present study extends the available evidence by evaluating this association in a clinically defined, chemotherapy-treated metastatic population reflecting routine practice in oncology.

Importantly, in our study, the association was not limited to the prespecified binary cut-off. Median OS progressively decreased across the conventional four CONUT categories, while continuous analysis showed that each one-point increase in CONUT was associated with shorter OS and PFS. Together, these findings support a graded association between increasing CONUT score and poorer outcomes. However, the very small number of patients in the highest CONUT category (9–12) warrants cautious interpretation of the category-specific survival estimates. In the present study, metastatic presentation (de novo vs. metachronous) was not independently associated with OS, and adjustment for this clinically relevant factor did not materially alter the association between elevated CONUT score and survival. The presence of comorbidity remained significantly associated with shorter OS in the multivariable analysis. Presence of comorbidity may negatively affect prognosis through multiple mechanisms, including reduced physiological reserve, impaired treatment tolerance, increased risk of treatment-related toxicity, and limited ability to receive intensive systemic therapy. Within the metastatic pancreatic cancer population, who are often elderly and nutritionally vulnerable, comorbid conditions may further aggravate functional decline and compromise overall clinical outcomes. Prior investigations similarly reported that higher presence of comorbidity is linked to worse survival and reduced treatment feasibility in pancreatic cancer populations [23,24,25].

Liver metastases remained significantly associated with shorter OS in the multivariable analysis. As the most common metastatic site in pancreatic adenocarcinoma, liver involvement often reflects advanced tumor burden and more aggressive disease biology. In addition, hepatic involvement may contribute to impaired metabolic homeostasis, systemic inflammation, cachexia, and deterioration in liver function, thereby negatively affecting treatment success and survival outcomes [26]. Previous studies have consistently reported poorer prognosis among metastatic pancreatic cancer patients with liver metastases compared with those without hepatic involvement [27].

Given the heterogeneity of first-line chemotherapy regimens, an exploratory comparison was performed between patients treated with gemcitabine plus nab-paclitaxel, the most frequently administered regimen, and those receiving all other regimens combined. No statistically significant difference in OS was observed between the groups. However, because treatment was not randomly assigned and the comparator group comprised several heterogeneous regimens, residual confounding related to treatment selection cannot be excluded.

This study has several limitations. Its retrospective design and the inclusion of only treatment-eligible patients with ECOG performance status 0–2 may limit the generalizability of the findings to the broader population of patients with metastatic pancreatic adenocarcinoma. Accordingly, these findings should not be extrapolated to patients who are unable to receive systemic chemotherapy, who may represent a clinically distinct population with different determinants of survival. In addition, baseline laboratory data required for CONUT calculation were unavailable for a small proportion of otherwise eligible patients, which may limit the generalizability of the findings. First-line chemotherapy regimens were heterogeneous, and treatment selection was influenced by patient- and disease-related characteristics; therefore, residual confounding related to treatment selection cannot be excluded. Histological grade data were unavailable for 83 patients (53.2%), largely because grading was not routinely reported in diagnostic biopsy specimens, and this variable was therefore not included in the analyses. Longitudinal changes in CONUT scores and formal assessments of cachexia were not available. Comorbidity was assessed as present or absent rather than using a validated index, precluding evaluation of the number and severity of comorbid conditions. Patients with metachronous metastatic disease may represent a nutritionally distinct subgroup, as prior pancreatic surgery may contribute to pancreatic exocrine insufficiency and malabsorption. However, these postoperative factors were not systematically documented, and residual confounding cannot be excluded. Nutritional interventions were not systematically recorded, and their potential effects on CONUT parameters and survival outcomes could not be assessed. Prospective studies are needed to validate these findings.

## 5. Conclusions

In this selected cohort of patients with metastatic pancreatic adenocarcinoma receiving first-line systemic chemotherapy, an elevated pretreatment CONUT score remained significantly associated with shorter OS after adjustment for selected clinical factors. Liver metastases and the presence of comorbidity were also associated with shorter OS. As a readily available laboratory-based index, CONUT may provide prognostic information; however, causality and clinical utility cannot be established from this retrospective study. Prospective validation is warranted.

## Figures and Tables

**Table 1:** Controlling Nutritional Status (CONUT) score components and classification.

Parameter	Normal	Mild	Moderate	Severe
Serum albumin (g/dL)	3.5–4.5	3.0–3.49	2.5–2.99	<2.5
Score	0	2	4	6
Total lymphocyte count (/mm^3^)	≥1600	1200–1599	800–1199	<800
Score	0	1	2	3
Total cholesterol (mg/dL)	≥180	140–179	100–139	<100
Score	0	1	2	3
CONUT score classification				
Total Score	0–1	2–4	5–8	9–12

CONUT: Controlling Nutritional Status score. The CONUT score = albumin score + total lymphocyte score + total cholesterol score.

**Table 2:** Distribution of baseline features across CONUT categories.

Characteristics	Total (n = 156, %)	CONUT 0–1 (n = 51, %)	CONUT ≥ 2 (n = 105, %)	*p* Value
Age, years (mean ± SD)	62.88 ± 10.51	64.29 ± 9.14	62.20 ± 11.09	0.244
BMI (kg/m^2^)	24.63 ± 5.05	24.63 ± 4.09	24.63 ± 5.06	0.997
Sex				
Female	66 (42.3%)	24 (47%)	42 (40%)	0.403
Male	90 (57.7%)	27 (53%)	63 (60%)	
ECOG PS				
0–1	137 (87.8%)	45 (88.2%)	92 (87.6%)	0.912
2	19 (12.2%)	6 (11.8%)	13 (12.4%)	
BMI (kg/m^2^)				
≤25	90 (57.7%)	31 (60.8%)	59 (56.2%)	0.586
>25	66 (42.3%)	20 (39.2%)	46 (43.8%)	
Metastases				
De novo	99 (63.5%)	35 (68.6%)	64 (61%)	0.350
Metachronous	57 (36.5%)	16 (31.4%)	41 (39%)	
Smoking				
Yes	76 (48.7%)	23 (45%)	53 (50.5%)	0.528
No	80 (51.3%)	28 (55%)	52 (49.5%)	
Alcohol				
Yes	25 (16%)	8 (15.7%)	17 (16.2%)	0.936
No	131 (84%)	43 (84.3%)	88 (83.8%)	
Presence of ≥1 comorbidity				
Yes	111 (71.2%)	36 (70.6%)	75 (71.4%)	0.913
No	45 (28.8%)	15 (29.4%)	30 (28.6%)	
Diabetes mellitus				
Yes	62 (39.7%)	23 (45.1%)	39 (37.2%)	0.341
No	94 (60.3%)	28 (54.9%)	66 (62.8%)	
Hypertension				
Yes	68 (43.6%)	25 (49%)	43 (41%)	0.341
No	88 (56.4%)	26 (51%)	62 (59%)	
Coronary artery disease				
Yes	27 (17.3%)	6 (11.8%)	21 (20%)	0.202
No	129 (82.7%)	45 (88.2%)	84 (80%)	
Family history				
Yes	28 (17.9%)	12 (23.5%)	16 (15.2%)	0.206
No	128 (82.1%)	39 (76.5%)	89 (84.8%)	
Weight loss				
Yes 5–10%	30 (17.2%)	10 (19.6%)	20 (19%)	0.020
Yes >10%	43 (27.6%)	7 (13.7%)	36 (34.3%)	
No	83 (53.2%)	34 (66.7%)	49 (46.7%)	
Loss of appetite				
Yes	71 (45.5%)	23 (45.1%)	48 (45.7%)	0.777
No	85 (54.5%)	28 (54.9%)	57 (54.3%)	
Localization				
Head	95 (60.9%)	26 (51%)	69 (65.7%)	0.077
Body/Tail	61 (39.1%)	25 (49%)	36 (34.3%)	
Prior curative-intent surgery				
Yes	55 (35.3%)	17 (33.3%)	38 (36.2%)	0.726
No	101 (64.7%)	34 (66.7%)	67 (63.8%)	
T Stage				
T1	6 (3.8%)	2 (3.9%)	4 (3.8%)	0.092
T2	37 (23.7%)	18 (35.3%)	19 (18.1%)	
T3	63 (40.4%)	16 (31.4%)	47 (44.7%)	
T4	50 (32.1%)	15 (29.4%)	35 (33.4%)	
N Stage				
N0	44 (28.2%)	12 (23.5%)	32 (30.5%)	
N1	54 (34.6%)	18 (35.3%)	36 (34.3%)	0.704
N2	58 (37.2%)	21 (41.2%)	37 (35.2%)	
Adjuvant chemotherapy				
Yes	54 (34.6%)	16 (31.4%)	38 (36.2%)	0.553
No	102 (65.4%)	35 (68.6%)	67 (63.8%)	
CEA *				
Normal	57 (36.5%)	14 (27.5%)	43 (41%)	0.106
High	99 (63.5%)	37 (72.5%)	62 (59%)	
CA 19-9 *				
Normal	37 (23.7%)	9 (17.7%)	28 (26.7%)	0.120
High	119 (76.3%)	42 (82.3%)	77 (73.3%)	
Liver metastases				
Yes	116 (74.4%)	39 (76.5%)	77 (73.3%)	0.674
No	40 (25.6%)	12 (23.5%)	28 (26.7%)	
Lymph node metastases				
Regional	74 (47.4%)	25 (49%)	49 (46.7%)	0.748
Non-regional	39 (25%)	12 (23.5%)	27 (25.7%)	
No	43 (27.6%)	14 (27.5%)	29 (27.6%)	
Peritoneal metastases				
Yes	36 (23.1%)	11 (21.6%)	25 (23.8%)	0.755
No	120 (76.9%)	40 (78.4%)	80 (76.2%)	
Lung metastases				
Yes	39 (25%)	11 (21.6%)	28 (26.7%)	0.493
No	117 (75%)	40 (78.4%)	77 (73.3%)	
Bone metastases				
Yes	10 (6.4%)	2 (4%)	8 (7.6%)	0.376
No	146 (93.6%)	49 (96%)	97 (92.4%)	
Metastatic organs				
1	57 (36.5%)	18 (35.3%)	39 (37.2%)	0.822
>1	99 (63.5%)	33 (64.7%)	66 (62.8%)	

* CEA levels ≤ 5 ng/mL and CA 19-9 levels ≤ 37 U/mL were considered normal; values above these cutoffs were considered elevated. BMI: Body Mass Index, ECOG PS: Eastern Cooperative Oncology Group Performance Status, CEA: Carcinoembryonic Antigen, CA 19-9: Carbohydrate antigen 19-9, CONUT: Controlling Nutritional Status.

**Table 3:** Findings from univariable and multivariable overall survival analyses.

Variables	Univariable Analysis HR (95% Cl)	*p*-Value	Multivariable Analysis HR (95%Cl)	*p* Value
Age	1.01 (0.99–1.03)	0.111	1.01 (0.99–1.02)	0.264
Male sex	0.95 (0.67–1.34)	0.778	1.02 (0.70–1.41)	0.914
BMI (<25 kg/m^2^)	1.17 (0.83–1.64)	0.367	-	-
ECOG PS 2	1.08 (0.64–1.83)	0.753	0.98 (0.56–1.72)	0.964
Metastatic presentation (metachronous)	1.02 (0.72–1.45)	0.892	1.24 (0.86–1.82)	0.236
Smoking	0.79 (0.57–1.11)	0.192	-	-
Alcohol	0.83 (0.53–1.32)	0.453	-	-
Presence of ≥1 comorbidity	1.67 (1.15–2.43)	0.007	1.88 (1.28–2.75)	0.001
Diabetes mellitus	1.22 (0.87–1.72)	0.242	-	-
Hypertension	1.33 (0.94–1.88)	0.105	-	-
Coronary artery disease	1.31 (0.85–2.02)	0.209	-	-
Weight loss	1.36 (0.88–2.21)	0.161	-	-
Loss of appetite	1.82 (0.25–13.20)	0.551	-	-
Prior curative-intent surgery	1.22 (0.85–1.75)	0.262	-	-
Adjuvant chemotherapy	1.21 (0.85–1.74)	0.281	-	-
T Stage (>T1)	0.95 (0.37–2.43)	0.926	-	-
N Stage (>N0)	0.89 (0.58–1.36)	0.605	-	-
CEA (high)	0.98 (0.69–1.40)	0.940	-	-
CA 19-9 (high)	1.29 (0.86–1.94)	0.212	-	-
Liver metastases	1.61 (1.08–2.41)	0.015	1.99 (1.31–2.03)	0.001
Lymph node metastases	1.38 (0.86–2.23)	0.180	-	-
Peritoneal metastases	0.98 (0.65–1.46)	0.933	-	-
Lung metastases	1.18 (0.80–1.74)	0.392	-	-
Bone metastases	1.13 (0.52–2.43)	0.748	-	-
Metastatic organs (>1)	1.06 (0.75–1.51)	0.708	-	-
Other first line regimens *	1.33 (0.90–1.98)	0.145	-	-
Elevated CONUT Score	1.92 (1.33–2.77)	<0.001	2.40 (1.63–3.52)	<0.001

* Gemcitabine plus nab-paclitaxel was the reference category. BMI: Body Mass Index, ECOG PS: Eastern Cooperative Oncology Group Performance Status, CEA: Carcinoembryonic Antigen, CA 19-9: Carbohydrate antigen 19-9, CONUT: Controlling Nutritional Status.

## Data Availability

The datasets generated and analyzed during the current study are not publicly available due to privacy and ethical considerations. However, they are available from the corresponding author upon reasonable request.

## Abbreviations

The following abbreviations are used in this manuscript:

CONUT	Controlling nutritional status
FOLFIRINOX	Fluorouracil, leucovorin, irinotecan, and oxaliplatin
OS	Overall survival
PFS	Progression-free survival
RECIST	Response evaluation criteria in solid tumors
ECOG PS	Eastern cooperative oncology group performance status
BMI	Body mass index
CEA	Carcinoembryonic antigen
